# Impact of cavotricuspid isthmus ablation for typical atrial flutter and heart failure in the elderly—results of a retrospective multi-center study

**DOI:** 10.3389/fcvm.2023.1109404

**Published:** 2023-04-17

**Authors:** Elke Boxhammer, Meriem Bellamine, Istvan Szendey, Mike Foresti, Marc Bonsels, Joseph Kletzer, Peter Jirak, Albert Topf, Johannes Kraus, Lukas Fiedler, Anna-Maria Dieplinger, Uta C. Hoppe, Bernhard Strohmer, Lars Eckardt, Rudin Pistulli, Lukas J. Motloch, Robert Larbig

**Affiliations:** ^1^ Clinic II for Internal Medicine, University Hospital Salzburg, Paracelsus Medical University, Salzburg, Austria; ^2^ Division of Cardiology, Hospital Maria Hilf Mönchengladbach, Mönchengladbach, Germany; ^3^ Clinic for Internal Medicine, Hospital Villach, Villach, Austria; ^4^ Department of Internal Medicine II, Wiener Neustadt Hospital, Wiener Neustadt, Austria; ^5^ Medical Faculty, Johannes Kepler University Linz, Linz, Austria; ^6^ Nursing Science Program, Institute for Nursing Science and Practice, Paracelsus Medical University, Salzburg, Austria; ^7^ Department of Cardiology II-Electrophysiology, University Hospital Muenster, Muenster, Germany; ^8^ Department of Cardiology I, Coronary and Peripheral Vascular Disease, Heart Failure, University Hospital Muenster, Muenster, Germany

**Keywords:** atrial flutter, cavotricuspid isthmus ablation, HFrEF, HFmrEF, heart failure

## Abstract

**Introduction:**

While in the CASTLE-AF trial, in patients with atrial fibrillation and heart failure with reduced ejection fraction, interventional therapy using pulmonary vein isolation was associated with outcome improvement, data on cavotricuspid isthmus ablation (CTIA) in atrial flutter (AFL) in the elderly is rare.

**Methods:**

We included 96 patients between 60 and 85 years with typical AFL and heart failure with reduced or mildly reduced ejection fraction (HFrEF/HFmrEF) treated in two medical centers. 48 patients underwent an electrophysiological study with CTIA, whereas 48 patients received rate or rhythm control and guideline-compliant heart failure therapy. Patients were followed up for 2 years, with emphasis on left ventricular ejection fraction (LVEF) over time. Primary endpoints were cardiovascular mortality and hospitalization for cardiac causes.

**Results:**

Patients with CTIA showed a significant increase in LVEF after 1 (*p* < 0.001) and 2 years (*p* < 0.001) in contrast to baseline LVEF. Improvement of LVEF in the CTIA group was associated with significantly lower 2-year mortality (*p* = 0.003). In the multivariate regression analysis, CTIA remained the relevant factor associated with LVEF improvement (HR: 2.845 CI:95% 1.044–7.755; *p* = 0.041). Elderly patients (≥ 70 years) further benefited from CTIA, since they showed a significantly reduced rehospitalization (*p* = 0.042) and mortality rate after 2 years (*p* = 0.013).

**Conclusions:**

CTIA in patients with typical AFL and HFrEF/HFmrEF was associated with significant improvement of LVEF and reduced mortality rates after 2 years. Patient age should not be a primary exclusion criterion for CTIA, since patients ≥70 years also seem to benefit from intervention in terms of mortality and hospitalization.

## 1. Introduction

With an incidence of around 88/100,000 person-years in the general population and 567/100,000 in patients over 80 years old, atrial flutter (AFL) is one of the most common arrhythmias (1). Electrophysiologically it is a macro-reentry circuit around the tricuspid annulus using the cavotricuspid isthmus (CTI) as a critical passage at the inferior boundary. Activation goes downward in the right atrium free wall, through the CTI, and ascends in the right septum (2, 3). AFL comes with many potentially severe complications, including atrial fibrillation (AF), AFL induced tachycardiomyopathy, heart failure (HF) and thromboembolic events leading to stroke (4–8). The pathophysiological mechanism of AFL consists of greatly increased atrial frequencies that lead to a consecutive overload of the atria, to a reduced filling of the atria and ultimately to a reduced ejection of blood volume from both the atria and the ventricles. Tachycardic conduction of AFL to the ventricles can induce tachymyopathy (1).

While initial treatment of AFL consists of rate and /or rhythm stabilization through medication and electrical or medical cardioversion, successful long-term treatment is often accomplished by catheter ablation. Although catheter ablation is considered safe, complications such as hematoma, hemorrhage or arteriovenous fistulae in the region of the inguinal access, the occurrence of pericardial effusion, or higher-grade AV block requiring a pacemaker are possible. These risks were analyzed by Steinbeck et al. with regard to the frequency of their occurrence in a nationwide in-hospital analysis (9). Overall, high success rates can especially be achieved in typical, CTI-dependent AFL due to its highly reproducible anatomical dependence (10–15) and ablation of the cavotricuspid isthmus (CTIA), respectively.

HF is one of the most frequent cardiac pathologies with high morbidity and mortality, especially in the older population, AFL itself might induce tachymyopathy leading to further deterioration of cardiac function. This pathology further promotes HF with reduced (HFrEF) or mildly reduced ejection fraction (HFmrEF). While extensive research has been conducted on the interplay between AF and HFrEF, similar studies on CTIA in the HF collective with AFL are limited. Some evidence suggests that CTIA is beneficial in patients with AFL and reduced left ventricular ejection fraction (LVEF), though more research is needed (1, 11, 13, 16, 17). Importantly, to the best of our knowledge, despite a high prevalence of HFrEF/HFmrEF in this population, outcomes in elderly patients with HFrEF/HFmrEF have not been investigated, yet. Of note, while this population is characterized by a pronounced age with a consequent high degree of multimorbidity, beneficial outcomes after CTIA in the follow-up are still a matter of debate.

Therefore, this study aimed to retrospectively investigate the effect of CTIA on left ventricular ejection fraction (LVEF) as well as follow-up outcomes including mortality and hospitalization rates in elderly patients with HFrEF/HFmrEF. We hypothesized, that despite the older age this intervention will improve LVEF with consequent reduction in hospitalization and mortality rates in this population.

## 2. Methods

### 2.1. Patient population

**Figure 1:**
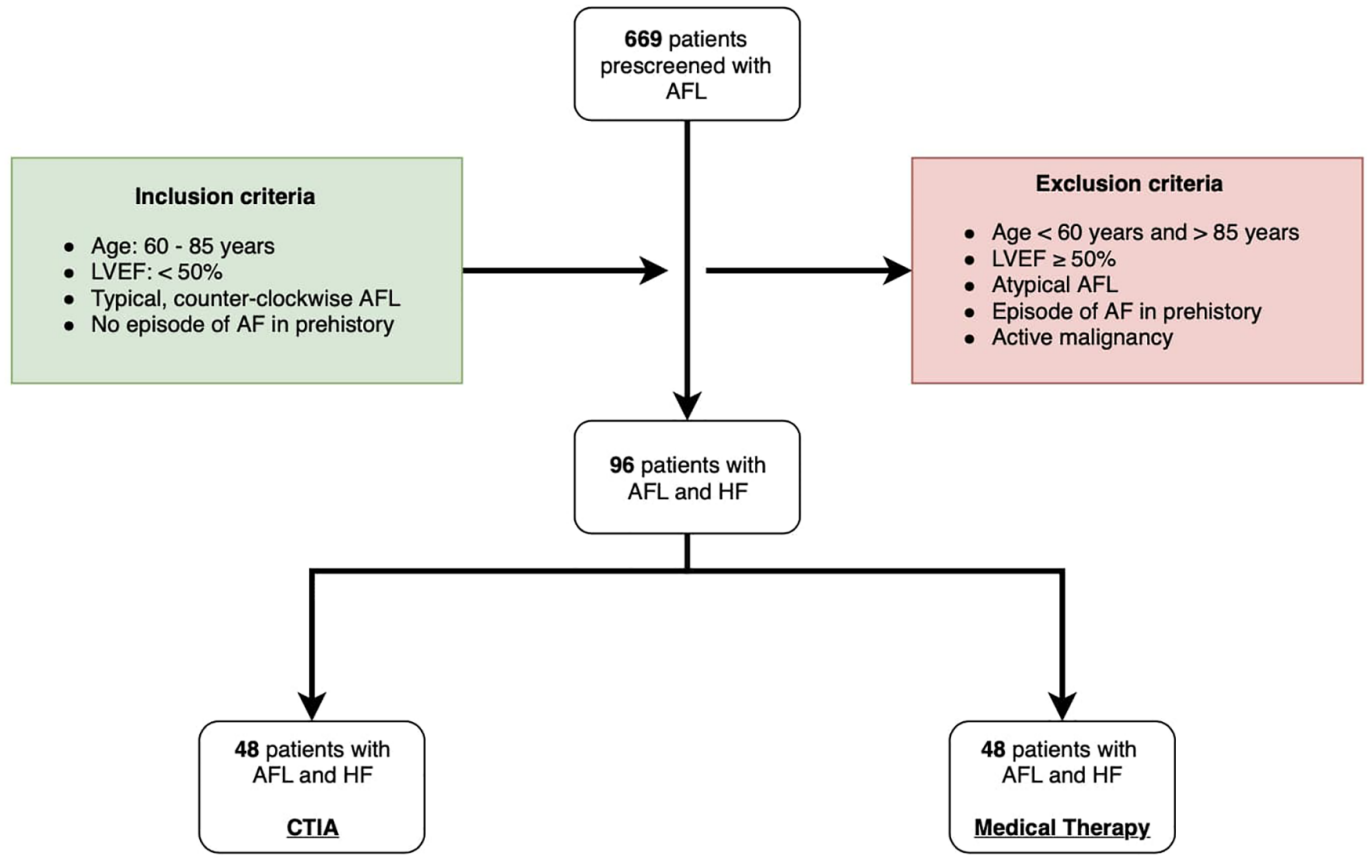
Flow chart of study design. AFL: atrial flutter; LVEF: left ventricular ejection fraction; AF: atrial fibrillation; HF: heart failure; CTIA: cavotricuspid isthmus ablation.

For this study, we retrospectively screened 669 consecutive patients (Figure 1) who were hospitalized due to symptomatic typical AFL (defined according to current guidelines with negative flutter waves in the inferior ECG leads (10) in two medical centers at the Paracelsus Medical University Hospital Salzburg and the Hospital Maria Hilf Mönchengladbach between 2010 and 2020. Inclusion criteria for this trial were symptomatic AFL with concomitant systolic HF according to HFrEF or HFmEF definition (defined as symptomatic HF with LVEF < 50%). Furthermore, since the trial aimed to focus on outcomes in the elderly, only patients with age 60–85 years were further analyzed. Cut off age of 85 years was chosen, to avoid scientifically relevant data bias, particularly regarding increased mortality and hospitalization rates in patients with pronounced age over 85 years. In addition, all patients with a prior relevant episode of AF and active malignancy were excluded. Thus, only data in patients with 60–85 years of age with a combination of relevant HFmrEF/HFrEF and isolated, typical, counter-clockwise AFL were included in the final analysis. We differentiated 3 different underlying pathologies of HF: Ischemic cardiomyopathy (ICM), dilated cardiomyopathy (DCM) and tachycardia-induced cardiomyopathy (t-CM). Choice of the treatment regime was based on current guidelines considering patients' comorbidities and also according to the will of the patient. Patients were also considered to be treated conservatively if no CTIA was performed for a follow-up period of 2 years after hospitalization for AFL. In the intervention group CTIA was performed within 6 months after hospitalization for AFL. Patients, who underwent CTIA beyond 6 months after hospitalization for AFL within 2 years of follow-up were also excluded from the study to avoid any potential bias caused by interventional treatment.

Based on our inclusion and exclusion criteria 96 were included into the study. 48 patients underwent CTIA ablation during the inclusion period and formed the ablation group while an additional 48 patients were treated conservatively and thus were included in the control group. In this group AFL was treated either by electrical cardioversion or medical treatment in order to achieve heart rate or rhythm control according to current guidelines (10). The study was approved by the ethical committee of the of Paracelsus Medical University Hospital Salzburg (Nr.: 415-E/247/9–2021) and the Ärztekammer North Rhine-Westphalia (Nr.:205/2021). Data analyses were performed at Paracelsus Medical University Hospital Salzburg and at Hospital Maria Hilf Mönchengladbach in accordance to principles of the Declaration of Helsinki and Good Clinical Practice. Written informed consent was available from all study participants.

### 2.2. Electrophysiological study and CTI ablation of AFL

Procedures were performed *via* femoral access under mild sedation and local anesthesia or general anesthesia in dependence of patient's request and hospital standards. Heparin was applied in a body weight-adapted manner (5,000–7,500 IE i.v.) regardless of the status of anticoagulation. CTIA aimed to interrupt the macro-reentrant atrial tachycardia and restore sinus rhythm. The ablation procedure was performed as previously described (18, 19). Typically, a four catheter study was carried out positioning a duo-decapolar catheter along the tricuspid valve anulus, a decapolar catheter in the coronary sinus, and a quadripolar catheter at the His position and in the right ventricle. In patients with atrial flutter at the time of ablation, the typical intracardiac ECG configurations could be detected with these catheters in the positions mentioned above. Patients with sinus rhythm during the intervention underwent pacing at the corona sinus ostium and at the infero-lateral portion of the right atrium *via* diagnostic catheters to verify isthmus conduction. In most cases, a steerable 7F ablation catheter was introduced *via* a long sheath (8F) for stability to deliver radiofrequency energy to the CTI. During the procedure, ablation lesions were placed point by point, starting at the tricuspid valve annulus, until the inferior vena cava was reached. It was at the discretion of each operator to add additional ablation lesions if previous ablation pulses did not achieve complete blockade of the CTIA. To verify the completeness of the ablation lesions that were set, detection of bidirectional block was performed, with stimulation from both sides of the “ablation line” (in the lower right atrium and coronary ostium). If blockade of excitation conduction was demonstrated in both directions, the procedure was considered successful (12). During this study (2010–2020), different ablation systems and techniques (with non-irrigated large tip electrodes or cooled tip ablation catheters) were used by experienced operators who performed at least ≥ 50 ablation procedures per year. After ablation, all patients received direct oral anticoagulation (DOAC) or a vitamin K antagonist for at least 3 months, after which they were reassessed for the need for DOAC/vitamin K antagonist therapy.

### 2.3. Medical treatment of AFL

Medical therapy was performed as outlined in current European Society for Cardiology (ESC)-guidelines (10). AFL primarily received permanent anticoagulation based on the same criteria as those with AF after exclusion of contraindications. The aim of the medical treatment was to achieve a ventricular rate of <100 beats per minute (bpm) as a resting rate. In this regard, beta-blockers or, in fewer cases, digitalis glycosides have been used preferentially. Alternatively, in symptomatic or hemodynamically unstable patients, electrical cardioversion was performed according to current guidelines. In some cases, antiarrhythmic drug therapy with class III or class Ic anti-arrhythmic drugs was established to maintain sinus rhythm.

### 2.4. Medical treatment of HF

Medical treatment of HF as well as AFL was performed according to the current ESC guidelines. Depending on relevant comorbidities and LVEF, patients received either an ACE inhibitor or an AT1 antagonist in combination with a beta blocker and an aldosterone antagonist. The respective dose was increased to maximum tolerability. In recent years, the ACE inhibitor or AT1 antagonist have been replaced by valsartan/sacubitril in patients with a HFrEF constellation. Drug therapy with an SGLT2 inhibitor is underrepresented in the current study because guideline recommendation of this medication for HFrEF was established just last year.

### 2.5. Follow up and primary endpoints

Patients were followed up for a total of two years. In addition to the in-hospital data, outpatient examinations of the respective hospital as well as examinations by general practitioners were used. In addition to the optimization of LVEF, death due to cardiovascular events and the hospitalization rate due to cardiac events were recorded as important primary endpoints. Causes of death due to accidents, malignancies, or other underlying diseases as well as elective reasons for hospitalization were excluded. Echocardiographically, LVEF was determined by applying the Simpson method. LVEF improvement in the follow-up was declared when LVEF recovered to ≥ 55% or an absolute increase in LVEF ≥ 30% was observed. This was in accordance with the criteria of Brembilla-Perrot et al. (21), as previously reported. Heart rhythm and also heart rate at the time of follow-up were determined firstly by a resting ECG and secondly by a 24 h ECG as an outpatient examination.

### 2.6. Statistical analysis

A statistical analysis was performed using SPSS 25 (SPSS Inc., Armonk, NY, United States).

At first, the Kolmogorov-Smirnov test was applied to test variables for normal distribution. Variables with nominal and ordinal scale level were specified as frequencies/percentages. Non-normally distributed, metric variables were presented as median with interquartile range (IQR).

For non-normally distribution of two groups (ablation group vs. medical treatment group) regarding metric variables Mann-Whitney-*U* test was performed and Chi-Square Test was calculated for nominal/ordinal variables. Friedman test with pairwise comparison was figured out to test dependent samples (LVEF change over time of ablation group or medical treatment group).

To identify possible influencing factors regarding the association between LVEF improvement and basic characteristics, a univariate, binary logistic regression analysis was completed. Subsequently, multivariate, binary logistic regression was performed to assess independent factors regarding the prediction of LVEF improvement. Therefore, covariates associated with detection of LVEF improvement in the univariate analysis (*p* = 0.100) were entered and a backward variable elimination was carried out.

Finally, Kaplan-Meier curves were created to analyze the probability of cardiovascular death and the probability of freedom from hospital admission after a follow-up period of 2 years.

A *p*-value of ≤ 0.050 was considered statistically significant.

## 3. Results

### 3.1. Patient characteristics

Between 2010 and 2020, a total of 96 patients were included at the Paracelsus Medical University Hospital Salzburg and the Hospital Maria Hilf Mönchengladbach (Figure 1). All patients had an isolated episode of typical AFL and an LVEF < 50% as determined by echocardiography using the Simpson method. 48 patients underwent ablation of the cavotricuspid isthmus (CTIA, ablation group), whereas an additional 48 patients were treated conservatively by maximal use of HF drug therapy or electrical cardioversion (medical therapy group). Immediate procedural success was achieved in 100% of all cases. Table 1 provides an overview of the baseline characteristics of the overall cohort and the two study groups. In the overall cohort, patients had an average age of 74.00 ± 11.50 years. Comorbidities were non-significantly distributed in the two groups. Regarding drug therapy, there was a significantly more frequent administration of valsartan/sacubitril and class III anti-arrhythmic drugs in the medical therapy group as compared to the ablation group (valsartan/sacubitril: 20.8% vs. 4.2%, *p* = 0.014; class III anti-arrhythmic drugs: 37.5% vs. 18.8%, *p* = 0.041). Concerning heart rate as well as heart rhythm, significant differences were observed at the time of study inclusion as more patients from the CTIA group were in sinus rhythm compared to medical therapy group (41.7% vs. 14.6%; *p* = 0.003) and consequently heart rate was significantly lower (75.00 ± 53 bpm vs. 112 ± 36.25 bpm; *p* = 0.005). At 1-year follow-up (Table 2), 72.9% of patients in the CTIA group and 83.3% of patients in the medical therapy group after exhaustion of conservative measures were also in sinus rhythm (*p* = 0.805). 18.8% developed atrial fibrillation within the first year after CTIA and during this period, AFL returned in 8.3%. Heart rate in 1-year follow up was not statistically significant at 70.00 ± 15.25 bpm in the CTIA group and 70 ± 22.00 bpm in the medical therapy group (*p* = 1.000).

**Table 1:** Baseline characteristics of overall cohort and different study groups (CTIA group vs. medical therapy group).

	Overall cohort *n* = 96	CTIA *n* = 48	Medical Therapy *n* = 48	*p*-value
**General characteristics**				
– Age—years (median ± IQR)	74.00 ± 11.50	71.00 ± 11.00	76.50 ± 9.25	0.101
– Sex male—*n* (%)	65 (67.7)	38 (79.2)	27 (56.3)	**0** **.** **016**
– Weight—kg (median ± IQR)	84.00 ± 20.00	84.00 ± 20.00	85.00 ± 25.00	0.633
**Cause of heart failure—*n* (%)**				
– Ischemic	51 (53.1)	23 (47.9)	28 (58.3)	0.306
– Dilated	30 (31.3)	16 (33.3)	14 (29.2)	0.660
– Tachycardia-induced	34 (35.4)	17 (35.4)	17 (35.4)	1.000
**Comorbidities—*n* (%)**				
– Arterial hypertension	79 (82.3)	39 (81.3)	40 (83.3)	0.789
– Hyperlipidemia	59 (61.5)	30 (62.5)	29 (60.4)	0.834
– Diabetes mellitus	36 (37.5)	16 (33.3)	20 (41.7)	0.399
– PAOD	10 (10.4)	4 (8.3)	6 (12.5)	0.504
– COPD	16 (16.7)	6 (12.5)	10 (20.8)	0.273
– Chronic kidney disease	26 (27.1)	12 (25.0)	14 (29.2)	0.646
– Myocarditis in pre-history	2 (2.1)	0 (0.0)	2 (4.2)	0.153
**Medication—*n* (%)**				
– Beta-blockers	89 (92.7)	43 (89.6)	46 (95.8)	0.239
– ACE/AT1-inhibitors	71 (74.0)	37 (77.1)	34 (70.8)	0.485
– Aldosterone-antagonists	33 (34.4)	18 (37.5)	15 (31.3)	0.519
– Ivabradine	1 (1.0)	0 (0.0)	1 (2.1)	0.315
– Valsartan/Sacubitril	12 (12.5)	2 (4.2)	10 (20.8)	**0** **.** **014**
– SGLT2	7 (7.3)	4 (8.3)	3 (6.3)	0.695
– Digitalis glucosides	1 (1.0)	0 (0.0)	1 (2.1)	0.315
– Class III anti-arrhythmic drugs	27 (28.1)	9 (18.8)	18 (37.5)	0.041
– Class Ic anti-arrhythmic drugs	5 (5.2)	3 (6.3)	2 (4.2)	0.646
**LVEF**				
– LVEF 0 years—% (median ± IQR)	35.00 ± 10.00	35.00 ± 10.00	35.00 ± 15.00	0.482
– HFmrEF—*n* (%)	40 (41.7)	24 (50.0)	16 (33.3)	0.098
– HFrEF—*n* (%)	56 (58.3)	24 (50.0)	32 (66.7)	0.098
**Other therapies—*n* (%)**				
– Electric cardioversion	37 (38.5)	24 (50.0)	13 (27.1)	0.026
– Rhythm control	14 (14.6)	6 (12.5)	8 (16.7)	0.563
– Rate control	90 (93.8)	43 (89.6)	47 (97.9)	0.092
**Rhythm and heart rate**				
– Sinus rhythm—*n* (%)	27 (28.1)	20 (41.7)	7 (14.6)	0.003
– AFL—*n* (%)	69 (71.9)	28 (58.3)	41 (85.4)	0.003
– Heart rate—bpm (median ± IQR)	100.00 ± 53.00	75.00 ± 53.00	112.00 ± 36.25	0.005
**General complications—*n* (%)**				
– Drug intolerance	4 (4.2)	2 (4.2)	2 (4.2)	1.000
**Complications of CTIA—*n* (%)**				
– Pacemaker	5 (5.2)	5 (10.4)	–	–
– Periprocedural complications	2 (2.1)	2 (4.2)	–	–

PAOD, peripheral arterial occlusive disease; COPD, chronic obstructive pulmonary diseases; LVEF, left ventricular ejection fraction; HFmrEF, heart failure with mildly-reduced ejection fraction; HFrEF, heart failure with reduced ejection fraction; CTIA, cavotricuspid isthmus ablation.

**Table 2:** Characteristics of overall cohort and different study groups (CTIA group vs. medical therapy group) concerning 1 and 2 year follow-up.

	Overall cohort *n*** **=** **96	CTIA *n*** **=** **48	Medical Therapy *n*** **=** **48	*p*-value
**LVEF**				
– LVEF 1 year—% (median ± IQR)	45.00 ± 20.00	50.00 ± 20.00	40.00 ± 16.25	< 0.001
– LVEF 2 years—% (median ± IQR)	50.00 ± 22.50	50.00 ± 20.00	42.50 ± 31.25	0.013
**Rhythm and heart rate**				
– Sinus rhythm 1 year—*n* (%)	75 (81.1)	35 (72.9)	40 (83.3)	0.805
– AFL 1 year—*n* (%)	5 (5.2)	4 (8.3)	1 (2.1)	0.454
– Heart rate 1 year—bpm (median ± IQR)	70.00 ± 18.50	70.00 ± 15.25	70.00 ± 22.00	1.000
– New onset of AF 1 year—*n* (%)	16 (16.7)	9 (18.8)	7 (14.6)	0.584
– New onset of AF 2 years—*n* (%)	22 (22.9)	13 (27.1)	9 (18.8)	0.502

LVEF, left ventricular ejection fraction; AFL, atrial flutter; AF, atrial fibrillation.

### 3.2. LVEF modifications with and without ablation

**Figure 2:**
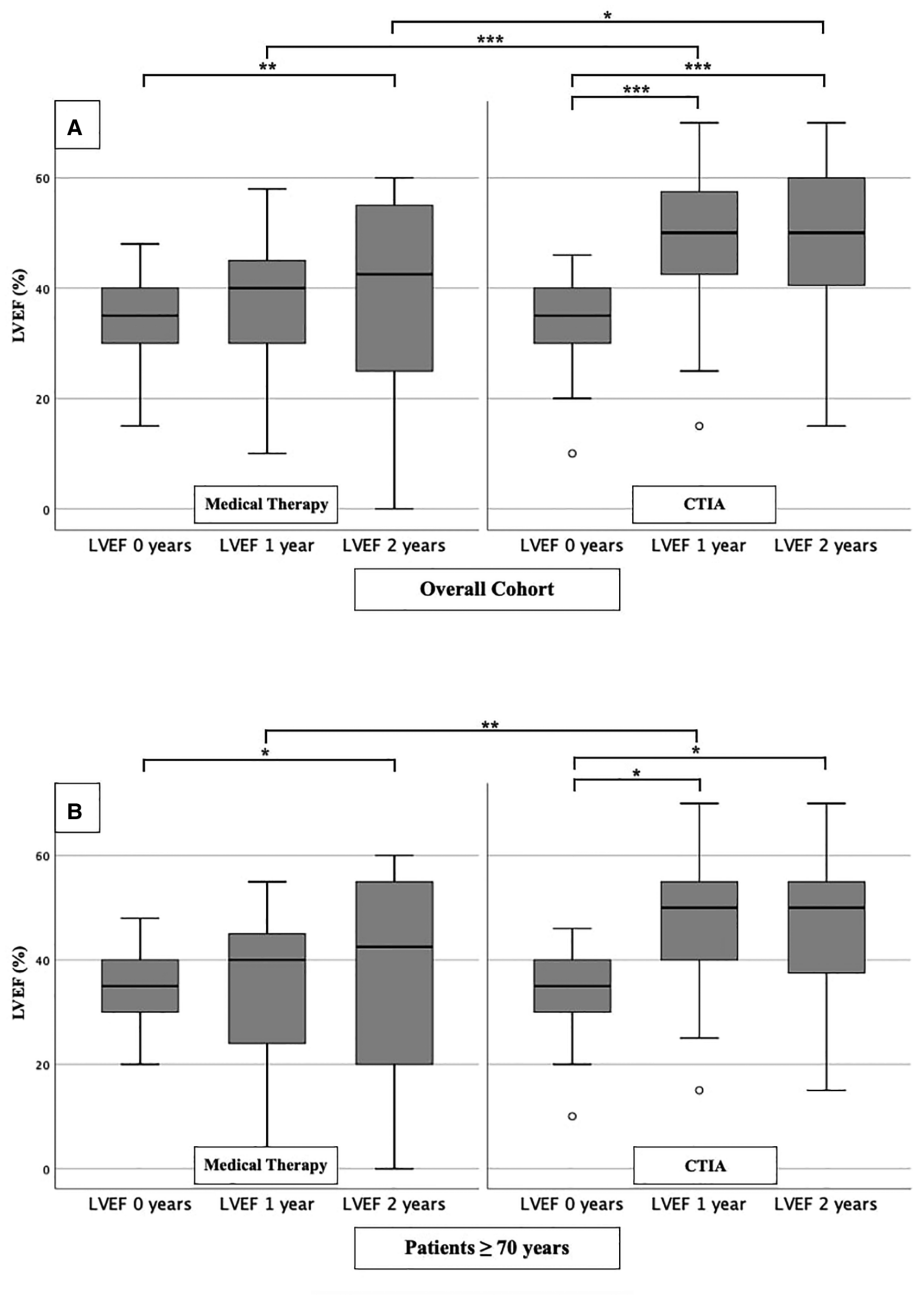
LVEF modifications in medical therapy group vs. ablation group of overall cohort. (**A**): All patients of overall cohort with unselected heart failure. (**B**): Patients ≥ 70 years of overall cohort with unselected heart failure. * *p* ≤ 0.050; ** *p* ≤ 0.010; *** *p* ≤ 0.001.

In order to analyze the impact of CTIA on LVEF, we could show a highly significant impact of CTIA on LVEF in the ablation group (*n* = 48) at 1 year (LVEF: 50.00 ± 20.00%; *p* < 0.001) and at 2 years (LVEF: 50.00 ± 20.00%; *p* < 0.001), respectively, compared with pre-intervention LVEF levels (LVEF: 35.00 ± 10.00%). In the medical therapy group (*n* = 48), we were able to demonstrate a significant improvement in LVEF only after 2 years (*p* = 0.010). On LVEF follow-up at 1 year (*p* < 0.001) and also at 2 years (*p* = 0.013), patients with ablation benefited significantly in terms of improved ejection fraction in contrast to the medical therapy group (Figure 2A). Additionally, we also examined patients ≥ 70 years of age to demonstrate the extent to which older patients also benefit from CTIA (Figure 2B). Here again, we were able to detect a significant improvement of LVEF in the ablation group (*n* = 29) after 1 year (LVEF: 50.00 ± 15.00%; *p* = 0.019) and after 2 years (LVEF: 50.00 ± 20.00%; *p* = 0.019) (baseline LVEF: 35.00 ± 10.00%). In the medical therapy group, as in the overall cohort, patients benefited only after 2 years (*p* = 0.037). The comparison of the two study groups with each other, showed a significant difference in LVEF at 1 year (*p* = 0.005).

**Figure 3:**
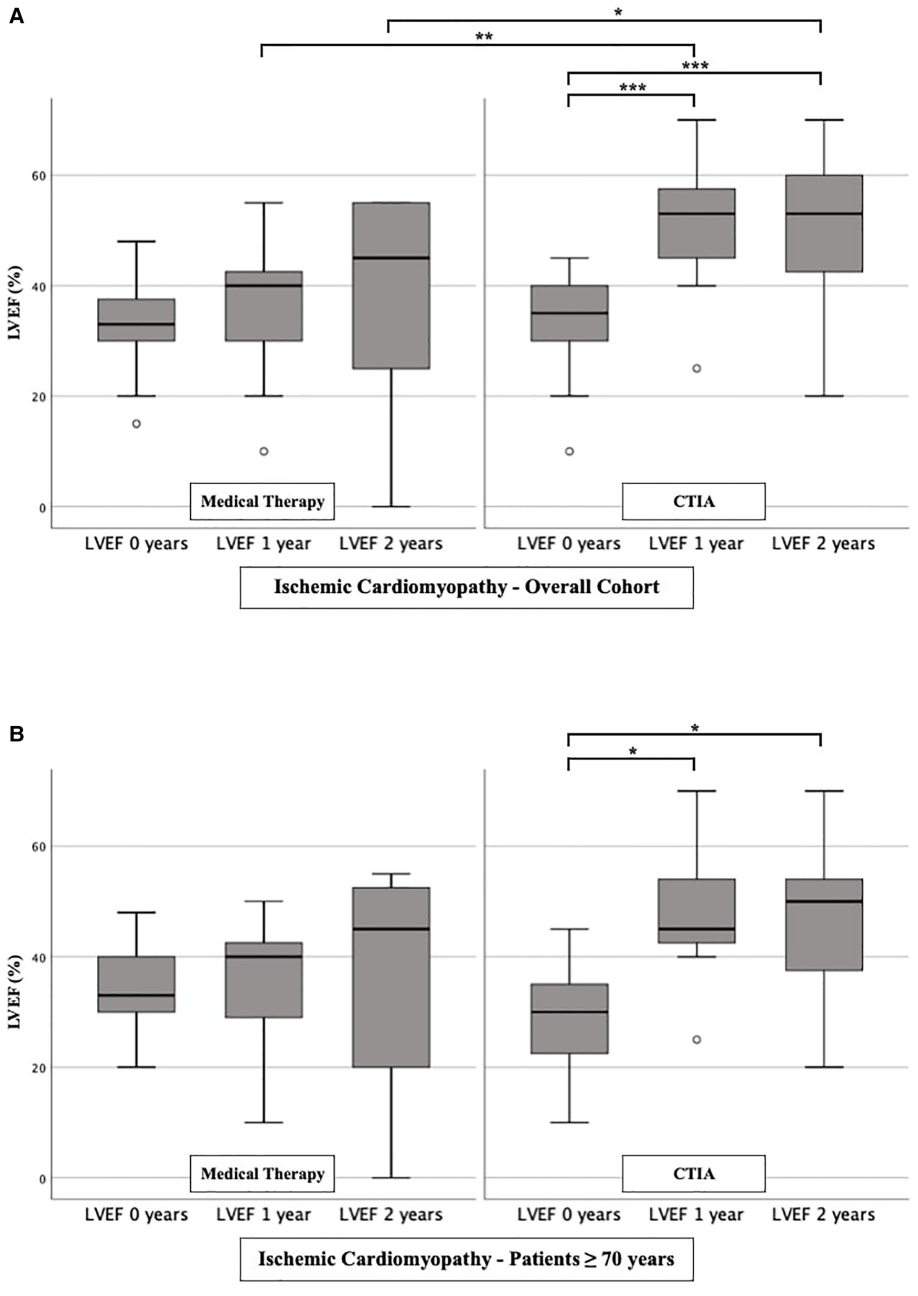
LVEF modifications in medical therapy group vs. ablation group of patients with ischemic cardiomyopathy. (**A**): All patients with ischemic cardiomyopathy. (**B**): Patients ≥ 70 years with ischemic cardiomyopathy. * *p* ≤ 0.050; ** *p* ≤ 0.010; *** *p* ≤ 0.001.

**Figure 4:**
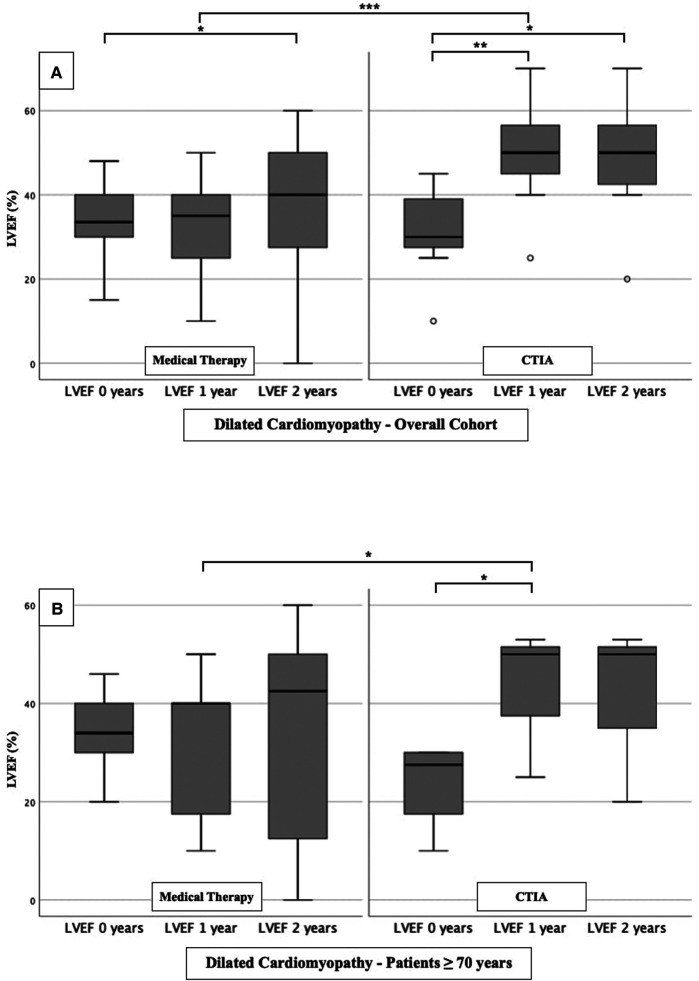
LVEF modifications in medical therapy group vs. ablation group of patients with dilated cardiomyopathy. (**A**): All patients with dilated cardiomyopathy. (**B**): Patients ≥ 70 years with dilated cardiomyopathy. * *p* ≤ 0.050; ** *p* ≤ 0.010; *** *p* ≤ 0.001.

**Figure 5:**
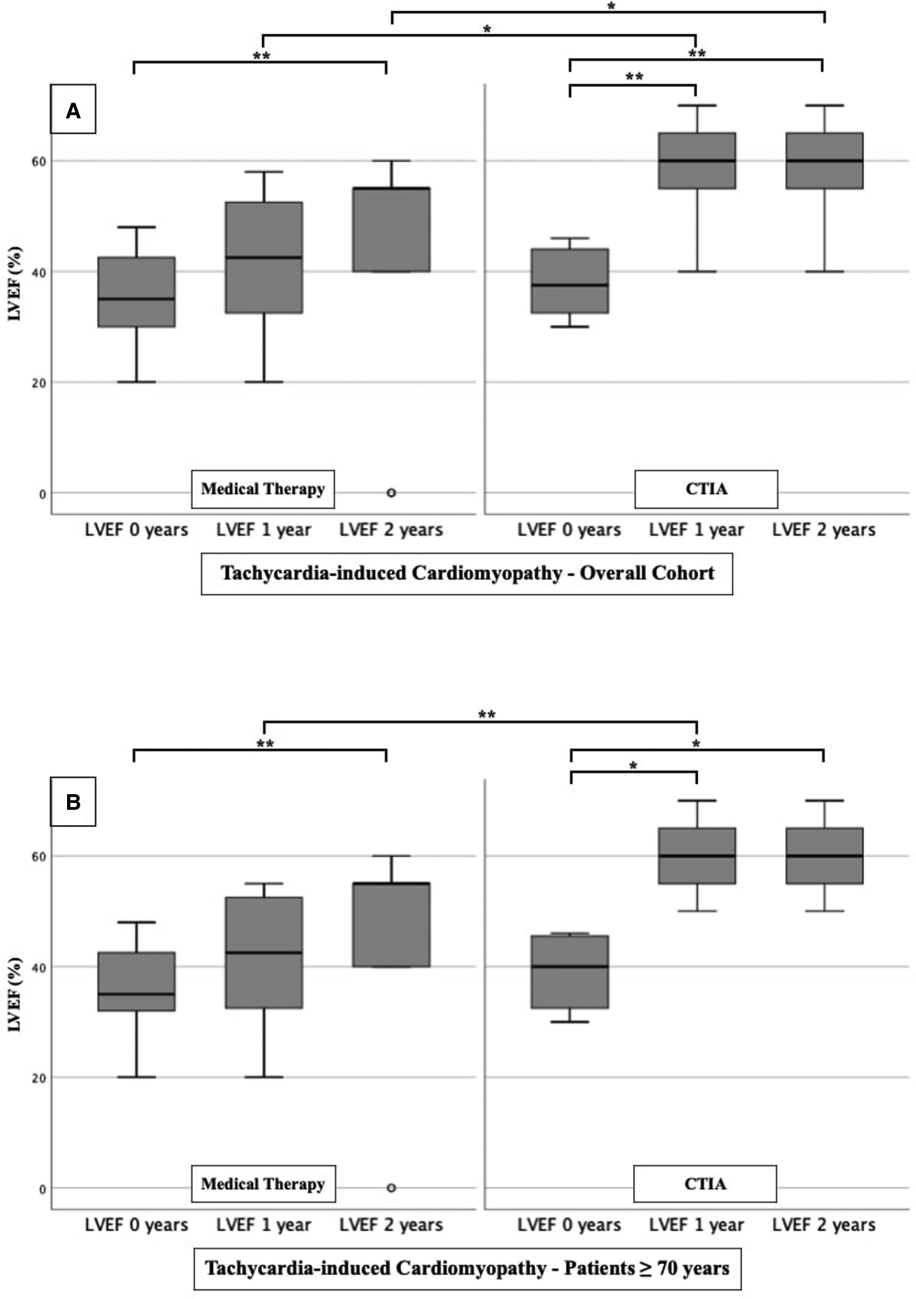
LVEF modifications in medical therapy group vs. ablation group of patients with tachycardia-induced cardiomyopathy. (**A**): All patients with tachycardia-induced cardiomyopathy. (**B**): Patients ≥ 70 years with tachycardia-induced cardiomyopathy. * *p* ≤ 0.050; ** *p* ≤ 0.010; *** *p* ≤ 0.001.

To further characterize the impact of CTIA on LVEF with regard to underlying pathologies we studied patients with ICM (Figure 3), DCM (Figure 4) and t-CM (Figure 5) separately. We also performed a subgroup analysis in elderly patients in each of these subgroups. In summary, for the respective overall cohorts of the various cardiomyopathies, we found similar results to those described above (Figures 3A, 4A, 5A). In detail, we detected a consistently and significant improvement of LVEF after 1 and 2 years of follow up in comparison to baseline LVEF in the ablation group. Also, in elderly patients (Figures 3B, 4B, 5B) with different types of cardiomyopathies, the respective ablation group showed significantly improved LVEF values at 1 year (ICM, DCM, t-CM) and at 2 years (ICM, t-CM) compared with baseline. Only in elderly patients with ischemic cardiomyopathy, we did not detect a significant impact of LVEF-improvement between ablation and medical therapy group (Figure 3B).

### 3.3. Criteria of LVEF improvement

In the multivariate regression analysis of overall cohort (Table 3), CTIA remained the relevant factor associated with LVEF improvement (HR: 2.845 95% CI 1.044–7.755; *p* = 0.041). The age ≥ 70 years was negatively associated with LVEF improvement (HR: 0.320 95% CI 0.119–0.866; *p* = 0.025). Although in the multivariate regression analysis, age was negatively associated with LVEF improvement (≥ 70 years) in the total study population. In contrast, in the separately examined regression analysis of ablated patients (Table 4), age ≥ 70 years was no longer statistically significant (HR 0.230 95% CI 0.049–1.088; *p* = 0.064).

**Table 3:** Tabular overview of univariate and multivariate binary logistic regression of overall cohort with regard to various clinical characteristics and LVEF improvement.

LVEF improvement Binary Logistic Regression	Univariate		Multivariate	
Overall Cohort (*n* = 96)	Hazard Ratio (95% CI)	*p*-value	Hazard Ratio (95% CI)	*p*-value
CTIA	3.276 (1.261–8.508)	0.015	2.845 (1.044–7.755)	0.041
Age ≥ 70 years	0.304 (0.199–0.771)	0.012	0.320 (0.119–0.866)	0.025
Sex (male)	0.656 (0.243–1.772)	0.406		
Weight	0.994 (0.970–1.019)	0.623		
Ischemic CMP	0.758 (0.311–1.848)	0.542		
Dilated CMP	0.700 (0.259–1.895)	0.483		
Tachycardia-induced CMP	1.709 (0.686–4.256)	0.250		
Arterial hypertension	2.036 (0.535–7.746)	0.297		
Hyperlipidemia	0.879 (0.354–2.183)	0.782		
Diabetes mellitus	0.483 (0.180–1.292)	0.147		
PAOD	0.256 (0.031–2.129)	0.208		
COPD	0.826 (0.241–2.829)	0.761		
Chronic kidney disease	0.700 (0.246–1.992)	0.504		
Beta-blockers	0.492 (0.103–2.363)	0.376		
ACE/AT1-antagonists	3.745 (1.018–13.777)	0.047	3.907 (0.999–15.276)	0.050
Aldosterone-antagonists	0.938 (0.366–2.403)	0.893		
Valsartan/Sacubitril	0.833 (0.208–3.345)	0.797		
SGLT2	1.024 (0.186–5.626)	0.978		
Class III anti-arrhythmic drugs	0.858 (0.314–2.344)	0.764		
Class Ic anti-arrhythmic drugs	1.760 (0.277–11.165)	0.549		
Electric cardioversion	0.566 (0.218–1.472)	0.243		
Medical rhythm control	2.179 (0.677–7.012)	0.192		
Medical rate control	0.364 (0.069–1.926)	0.234		
New onset of atrial fibrillation	0.806 (0.274–2.369)	0.695		
Recurrence of atrial flutter	0.297 (0.035–2.548)	0.268		

CTIA, cavotricuspid isthmus ablation; CMP, cardiomyopathy; PAOD, peripheral arterial occlusive disease; COPD, chronic obstructive pulmonary diseases.

**Table 4:** Tabular overview of univariate and multivariate binary logistic regression of CTIA group with regard to various clinical characteristics and LVEF improvement.

LVEF improvement Binary Logistic Regression	Univariate		Multivariate	
CTIA (*n* = 48)	Hazard Ratio (95% CI)	*p*-value	Hazard Ratio (95% CI)	*p*-value
Age ≥ 70 years	0.277 (0.082–0.940)	0.039	0.230 (0.049–1.088)	0.064
Sex (male)	1.714 (0.421–6.979)	0.452		
Weight	0.980 (0.943–1.019)	0.308		
Ischemic CMP	1.368 (0.429–4.364)	0.597		
Dilated CMP	0.877 (0.255–3.011)	0.835		
Tachycardia-induced CMP	1.616 (0.485–5.384)	0.434		
Arterial hypertension	2.705 (0.497–14.718)	0.250		
Hyperlipidemia	0.724 (0.220–2.378)	0.594		
Diabetes mellitus	0.378 (0.100–1.425)	0.151		
PAOD	0.481 (0.046–5.006)	0.541		
COPD	0.735 (0.121–4.474)	0.739		
Chronic kidney disease	1.769 (0.473–6.624)	0.397		
Beta-blockers	0.395 (0.060–2.623)	0.336		
ACE/AT1-antagonists	9.474 (1.099–81.684)	0.041	15.943 (1.271–200.049)	0.032
Aldosterone-antagonists	0.955 (0.289–3.158)	0.939		
SGLT2	1.588 (0.204–12.359)	0.659		
Class III anti-arrhythmic drugs	2.232 (0.514–9.694)	0.284		
Class Ic anti-arrhythmic drugs	0.750 (0.063–8.897)	0.820		
Electric cardioversion	0.120 (0.031–0.465)	0.002	0.113 (0.024–0.530)	0.006
Medical rhythm control	10.000 (1.064–94.012)	0.044	2.633 (0.217–31.898)	0.447
Medical rate control	0.395 (0.060–2.623)	0.336		
New onset of atrial fibrillation	0.571 (0.145–2.247)	0.423		
Recurrence of atrial flutter	0.471 (0.045–4.919)	0.529		

CTIA, cavotricuspid isthmus ablation; CMP, cardiomyopathy; PAOD, peripheral arterial occlusive disease; COPD, chronic obstructive pulmonary diseases.

### 3.4. Hospital admission and mortality with and without ablation

**Figure 6:**
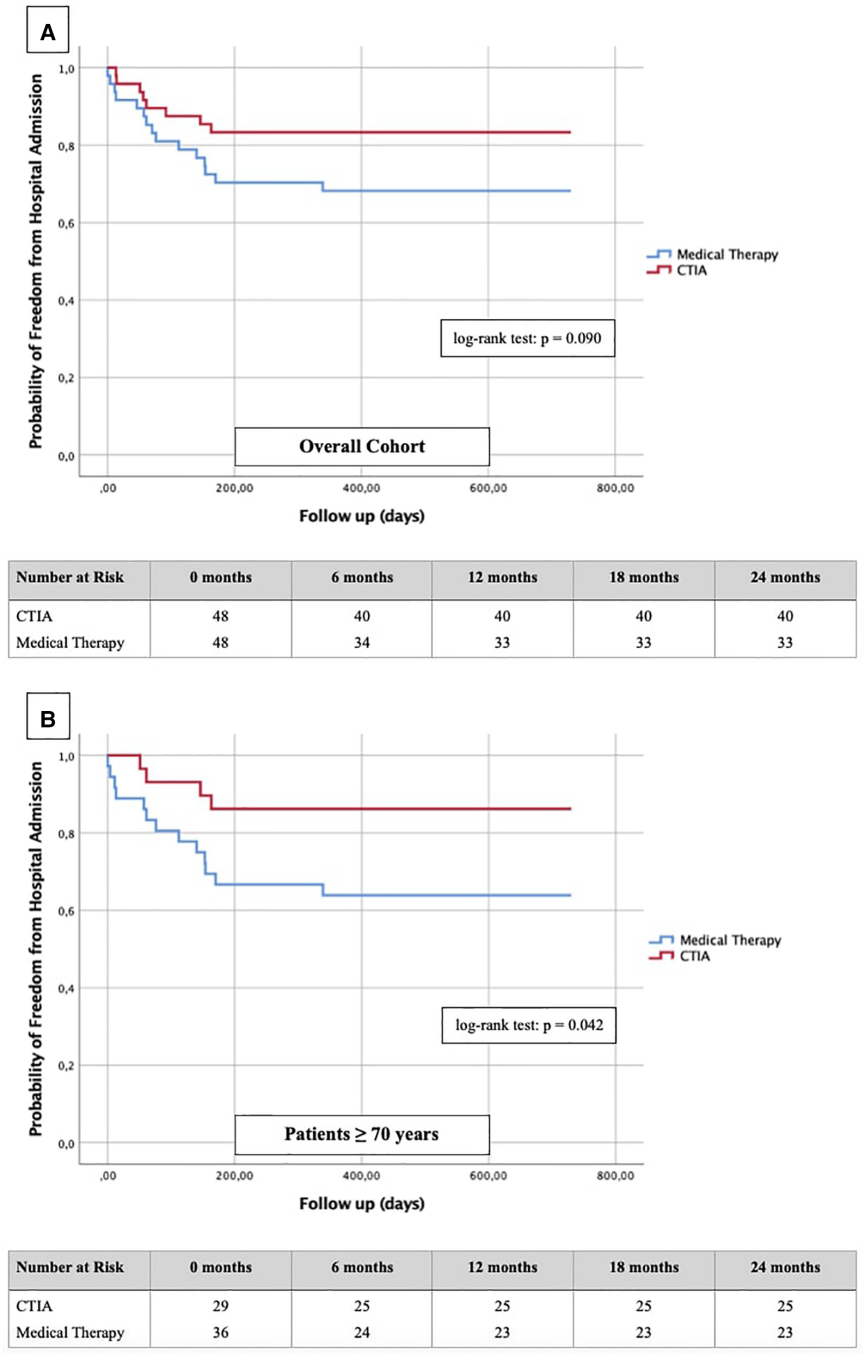
Kaplan-Meier curves with corresponding numbers at risk for detection of 2-year re-hospitalization rate regarding cardiovascular events in dependence of ablation vs. medical therapy.

**Figure 7:**
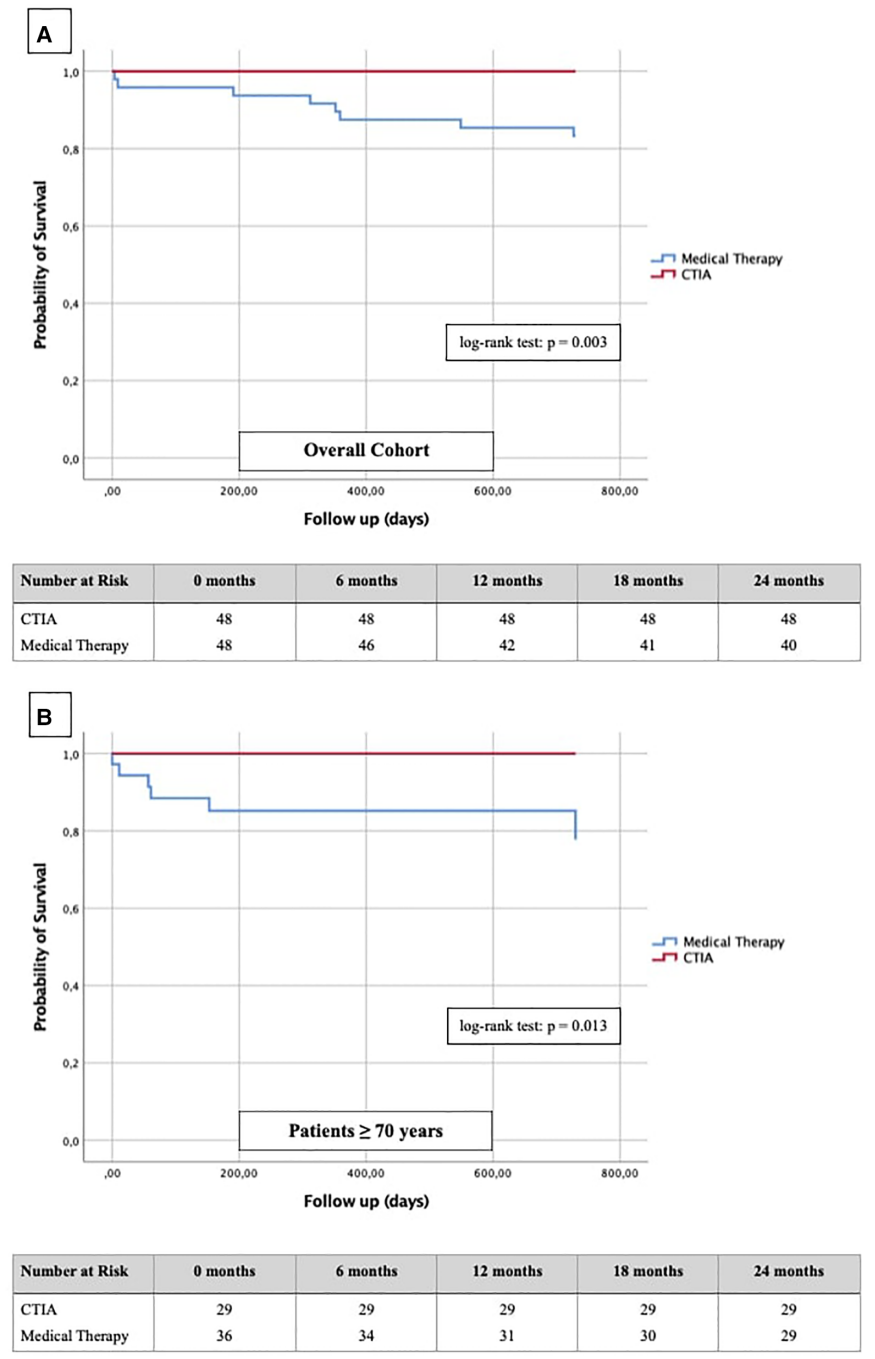
Kaplan-Meier curves with corresponding numbers at risk for detection of 2-year survival regarding cardiovascular death in dependence of ablation vs. medical therapy.

To illustrate the impact of CTIA on relevant clinical endpoints such as cardiovascular hospital admission (Figure 6) and mortality (Figure 7) we created Kaplan-Meier curves of the overall cohort and the elderly. We found a significant reduction of hospital admission in elderly patients (*n* = 64; *p* = 0.042) while we could not reach statistical significance in the overall cohort regarding this endpoint (*n* = 96; *p* = 0.090).

In terms of mortality, we found CTIA to have a highly significant impact vs. medical therapy alone in the overall cohort (*n* = 96; *p* = 0.003). Also, we found a similar effect with a highly significant benefit of elderly patients from CTIA vs. medical therapy alone (*n* = 64; *p* = 0.013).

## 4. Discussion

Many studies, most notably the CASTLE-AF trial published in 2018 (17), have already investigated the association between HF and AF. However, very few studies to date have analyzed typical, counter-clockwise AFL as a cause of new-onset HF or as a reason for worsening of a preexisting cardiomyopathy (1). Additionally, in daily practice, many elderly patients with typical AFL are not treated with ablation since it is an invasive procedure, patients tend to be increasingly frail and the clinical benefit of CTIA in this subgroup is unknown. Also, the study by Brembilla-Perrot et al. (20) described that a failed recovery of LVEF after ablation may also be due to an advanced patient age. Therefore, the aim of this study was to analyze primarily the clinical outcomes of elderly patients with ≥ 70 years of age, impaired LVEF and episodes of typical counter-clockwise AFL. In detail, we focused on describing the impact of LVEF changes after ablation in comparison to drug therapy and electrical cardioversion. Additionally, we analyzed the influence of ablation on the endpoints of cardiovascular hospitalization and mortality. We used a highly preselected cohort of 96 study patients (Figure 1) from two large hospitals in Germany and Austria to address these issues.

At first, we were able to detect a highly significant improvement of LVEF after CTIA vs. medical therapy within up to 1 year of follow-up. We observed this effect regardless of the genesis of heart failure. This underlines that CTIA is a highly effective treatment of AFL and concomitant HF (21). Similar effects of CTIA on LVEF in AFL-related tachymyopathy have already been described in other studies (13, 20, 22–24). In these studies, the LVEF response to CTI ablation was consistently very good (57%–100% of the respective cohort) (1). We observed a slightly lower percentage of 47.1% (data not shown), which might be due to the age of our collective and the fact that we included patients with ICM, DCM and t-CM. However, tachycardia-induced cardiomyopathy was not an isolated criterion for LVEF optimization in binary logistic regression analysis in either the overall cohort or the ablation cohort. Interestingly, despite other studies which described ICM or DCM as a reason for failed recovery of LVEF after CTIA (10, 25), we were able to detect an improvement of LVEF in our subgroup analyses in ICM and DCM as well (Figures 3A,B and Figures 4A,B). This may be due, among other reasons, to the fact that patients were sometimes not strictly dividable into one cardiomyopathy group.

In order to further describe the potential benefits of CTIA, we also analyzed our data with regard to relevant endpoints such as hospitalization rate for cardiovascular events and cardiovascular mortality. Dewland et al. (26) already reported on healthcare utilization and clinical outcomes after catheter ablation of AFL. These authors analyzed an unselected patient population (patients with and without HF) and found CTIA to be associated with a significantly reduced rate of hospitalization. A small cohort (*n* = 79) from New Zealand by Foo et al. (27) included patients with AF/AFL and concomitant HF (LVEF ≤ 40%) of any etiology. 47 patients had AFL, and the rate of rehospitalization for HF and rhythmological events (secondary outcomes) was significantly higher after electrical cardioversion than after ablation. In our study, successful CTIA did not result in a significantly lower hospitalization rate of the entire cohort compared to medical therapy alone, but nevertheless showed a relevant tendency to decrease (Figure 6A). In our subgroup analysis of elderly patients however, we were able to detect a significant reduction of hospitalization (Figure 6B, *p* = 0.042). In summary, we hypothesize that CTIA does have a relevant impact on cardiovascular hospitalization, but larger, prospective and randomized studies need to confirm this. Our study also suffered from methodical limitations regarding this endpoint since cardiovascular hospitalization in other hospitals were not always documented in the available databases or communicated to the participating general practitioners.

Finally, regarding the impact of CTIA on cardiovascular mortality Yugo et al. (28) reported a significantly reduced mortality in a long-term follow-up in patients with typical AFL independent of any HF after CTI ablation. Thakkar et al. (29) showed similar ratios in AFL patients with HFpEF constellation, whereas Jani et al. (30) demonstrated statistically significantly better survival within 1 year after CTI ablation only in HFrEF and not in HFpEF patients. Our results in HFrEF and HFmrEF patients fit seamlessly into the previous data (Figure 7A). Additionally, we clearly show for the first time that also elderly patients have a significantly better survival after CTIA as compared with medical therapy (Figure 7B, *p* = 0.013). The clinician should be aware of this finding when considering these patients for ablation or not. Ultimately, at the current state of research, it is an individual decision of the interventionalist to perform CTI ablation on elderly patients after a thorough risk-benefit assessment. Larger prospective multi-center studies should be performed to clarify this.

**Figure 8:**
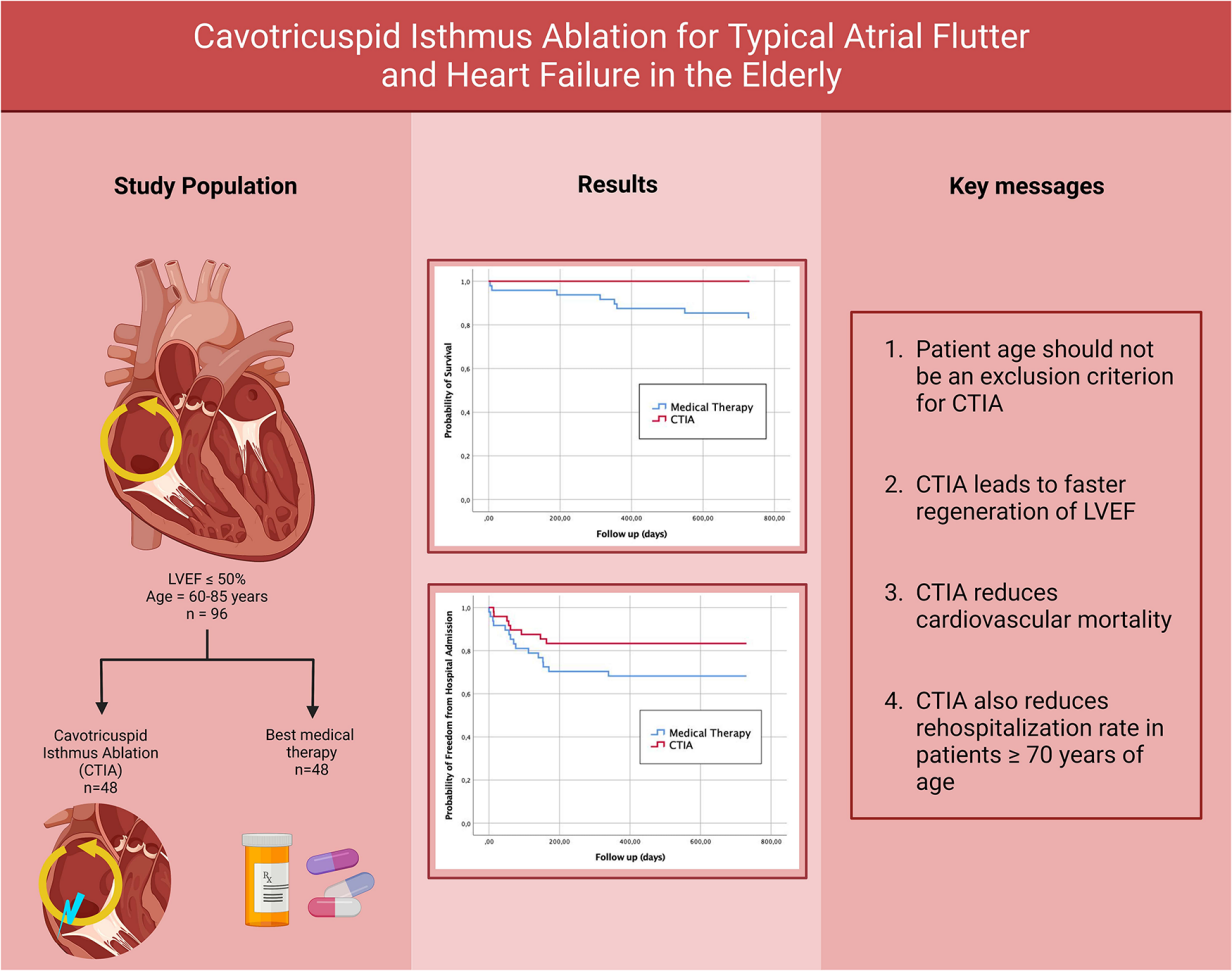
Graphical abstract of the study (created with bioRender.com).

A graphical abstract providing an overview of the methodology, the relevant results and the key messages can be found in Figure 8.

## 5. Limitations

The most obvious limiting factor of this study is its retrospective design. Additionally, it should be mentioned that the study population (*n* = 96) is relatively small for a retrospective study. However, this can be attributed to the strict pre-selection of patients included in this study.

## 6. Conclusion

CTIA in patients with typical AFL and HF was associated with significant optimization of LVEF and lower rates of hospitalization and mortality after 2 years. Patient age should not be a primary exclusion criterion for CTIA because patients ≥ 70 years also benefited significantly from intervention in terms of mortality, hospitalization and LVEF optimization.

## Data Availability

The raw data supporting the conclusions of this article will be made available by the authors, without undue reservation.
